# Therapeutic Potential and Mechanistic Pathways of Plant-Based Supplements and Exercise Training in Blood Pressure Management: An Evidence-Based Review and Research Agenda

**DOI:** 10.3390/nu18040700

**Published:** 2026-02-22

**Authors:** Behzad Taati, Georgian Badicu, Jolita Vveinhardt

**Affiliations:** 1 Independent Researcher, Rasht 4198713433, Guilan, Iran; 2 Department of Physical Education and Special Motricity, Faculty of Physical Education and Mountain Sports, Transilvania University of Braşov, 500068 Braşov, Romania; georgian.badicu@unitbv.ro; 3 Klaipėdos Valstybinė Kolegija/Higher Education Institution, 91274 Klaipėda, Lithuania

**Keywords:** physical activity, resistance training, aerobic training, beetroot, green tea, garlic, curcumin, resveratrol

## Abstract

Hypertension (HTN) remains a leading modifiable risk factor for cardiovascular disease, and non-pharmacological strategies combining exercise training with plant-derived bioactive supplementation are increasingly recognized as promising adjuncts for blood pressure (BP) management. This evidence-based review synthesizes findings from 31 clinical studies investigating selected plant-based supplements with the strongest available clinical evidence, namely beetroot juice (BRJ), green tea (GT), curcumin (CN), resveratrol (RSV), and garlic, administered alone or in combination with different exercise modalities across acute, short-term, and long-term interventions. Collectively, the evidence indicates that BRJ exerts the most consistent BP-lowering effects, particularly during aerobic training performed at ~50% heart rate reserve (HRR), or ~60% peak oxygen consumption (VO_2peak_) in individuals with early-stage vascular dysfunction. CN and garlic also enhance exercise-induced BP reductions, especially in older or metabolically compromised populations. GT shows variable outcomes depending on caffeine content, exercise modality, and participant health status, while RSV provides modest vascular support, often contingent on concurrent training. Mechanistically, these botanicals and exercise converge on key vascular-regulatory pathways, including enhanced nitric oxide (NO) bioavailability, reduced oxidative stress and inflammation, attenuated renin–angiotensin–aldosterone system (RAAS) and sympathetic activity, and improved mitochondrial function through Sirtuin 1 (SIRT1)/AMP-activated protein kinase (AMPK) signaling. Together, these integrated mechanisms improve endothelial function, lower vascular resistance, and ultimately reduce BP. From a translational standpoint, combining exercise with targeted plant-based supplementation offers a safe, accessible, and physiologically synergistic strategy for BP control in clinical populations. Future research should define optimal dosing, timing relative to exercise, and population-specific efficacy to inform precision-based, integrative interventions for HTN management.

## 1. Introduction

Hypertension (HTN) is a leading modifiable risk factor for cardiovascular morbidity and mortality worldwide, affecting over one billion adults [1]. Most cardiovascular risk factors can be prevented or managed through lifestyle modifications [2], particularly nutritional and exercise interventions [3,4]. Regular exercise training and healthy dietary habits have consistently been shown to lower arterial blood pressure (BP) and improve vascular function [2,4,5,6,7,8].

Beyond traditional approaches, growing evidence indicates that plant-based bioactive compounds (e.g., polyphenols, flavonoids, and organosulfur compounds) may exert antihypertensive effects through mechanisms including enhanced nitric oxide (NO) bioavailability, improved endothelial function, and reduced oxidative stress [9,10,11,12,13,14]. However, despite widespread use, the efficacy of many plant-derived supplements remains uncertain, and results across clinical studies are inconsistent [15]. Consequently, identifying combinations of nutritional supplementation and exercise training that produce additive or synergistic BP-lowering effects represents a promising research direction.

Recent experimental findings suggest that combining naturally derived supplements with exercise training may produce additive or synergistic BP-lowering effects [4,16,17]. Nevertheless, comprehensive synthesis of this emerging evidence is lacking. Therefore, this review aims to summarize current clinical findings on the effects of selected plant-based supplements, administered alone or in combination with exercise training, on BP regulation in clinical populations. It should be noted that this review was designed as a narrative evidence-based synthesis rather than a formal systematic review or meta-analysis. Accordingly, no predefined PRISMA framework or statistical pooling procedures were applied. This approach allows integration of mechanistic evidence with clinical findings and supports the identification of knowledge gaps to inform future research directions in hypertension management.

## 2. Study Overview

This review included studies examining the acute (30–180 min prior to exercise), short-term (3 days–3 weeks), and long-term (4–16 weeks) effects of combined plant-based supplementation and exercise training on BP responses and adaptations in clinical populations. As shown in Table 1, a total of 31 original studies were identified across five commonly studied herbal supplements: beetroot juice (BRJ, *n* = 14), green tea (GT, *n* = 6), curcumin (CN, *n* = 4), resveratrol (RSV, *n* = 3), and garlic (*n* = 3). All studies were conducted in human participants with various clinical conditions, including pre-HTN or HTN, metabolic syndrome (MetS), obesity, type 2 diabetes mellitus (T2DM), heart failure, peripheral artery disease (PAD), coronary artery disease (CAD), and chronic obstructive pulmonary disease (COPD).

Most studies employed a randomized controlled trial (RCT) design, with crossover or parallel-group allocation. Double-blind, placebo-controlled methods were reported in the majority of acute and short-term trials [24,31,32], while quasi-experimental designs with parallel groups were more common among long-term interventions [40,41]. Interventions typically compared herbal supplementation combined with exercise versus exercise alone, supplementation alone, or placebo/control.

Sample sizes ranged from 8 to 81 participants per study, with group sizes between 8 and 20 participants per arm. The participants were mainly middle-aged and older adults, both male and female. Only a few studies focused exclusively on women [32,37] or men [33].

Herbal supplement doses varied widely depending on the compound. For instance, BRJ doses ranged from 70 to 500 mL per day, GT from 100 mg capsules to 3 g brewed leaves per day, curcumin from 10.5 to 80 mg/day, RSV from 300 to 500 mg/day, and garlic 1000 mg capsules daily. Furthermore, exercise protocols also varied. Aerobic training (AT) ranged from moderate-intensity continuous walking/jogging [(e.g., 60–85% maximum heart rate (HR_max_)] to high-intensity interval training (HIIT, e.g., 90–95% HR_max_). Resistance training (RT) included dynamic low-intensity [50% of one-repetition maximum (1RM)] circuits and high-resistance [80% of peak power (P_peak_)], isometric, and eccentric training programs. The acute and short-term studies assessed BP changes during the exercise or recovery period, whereas most long-term studies evaluated resting or ambulatory BP adaptations.

## 3. Beetroot

Beetroot (*Beta vulgaris*) is recognized as an ergogenic and multifunctional supplement with potential benefits for vascular dysfunction, atherosclerosis, cardiorespiratory disorders, and diabetes [43]. Its high inorganic nitrate (NO_3_^−^) content may enhance NO bioavailability, supporting vascular function and compensating for NO-impaired pathways in HTN [44]. In addition, beetroot is rich in other bioactive phytochemicals, indicating potential BP-lowering effects beyond nitrate-dependent mechanisms [10].

We identified 14 studies investigating the effects of BRJ on BP responses to exercise in clinical populations. These include seven acute studies [18,19,20,21,22,23,24], four short-term supplementation studies [27,28,29,30], two studies investigating both acute and short-term supplementation effect [25,26], and one long-term combined intervention [7].

### 3.1. Acute BRJ Effects on BP Responses to Exercise

Acute BRJ supplementation (typically a single dose of 70–500 mL providing ~6–13 mmol NO_3_^−^, administered 30–180 min prior to exercise) has been examined in nine studies involving diverse clinical populations. Most studies involved AT and reported significant increases in plasma NO_3_^−^ and nitrite (NO_2_^−^) concentrations after BRJ ingestion, confirming bioavailability, but BP responses were variable.

In AT settings, acute BP-lowering effects of BRJ has been demonstrated in several studies. For instance, Benjamim et al. [26] reported a significant reduction in systolic BP (SBP) and mean arterial pressure (MAP) 50 min post-exercise in hypertensive postmenopausal women following acute BRJ intake compared with placebo. Similarly, Kenjale et al. [18] found significant decreases in diastolic BP (DBP) during a graded cardiopulmonary exercise test in patients with PAD after BRJ supplementation. Berry et al. [19] also observed significant reductions in exercise DBP in patients with COPD, while Bezerra et al. [21] demonstrated that acute BRJ supplementation significantly reduced ambulatory SBP up to six hours post-exercise [running at 50% heart rate reserve (HRR)] in obese individuals.

However, other studies reported null effects despite increased NO_3_^−^ and NO_2_^−^ levels. Amaral et al. [20] found no changes in BP during the recovery period after moderate-intensity AT (i.e., 65–70% HRR) in hypertensive postmenopausal women, and van der Avoort et al. [22] similarly reported no significant BP changes in PAD patients. Eggebeen et al. [25] and Gonzaga et al. [23] also found no significant effects on BP or heart rate variability in patients with heart failure and CAD, respectively.

While only one study examined the acute BP responses to RT, a similar pattern was observed. In drug-naïve hypertensive individuals performing isometric handgrip exercise, Zafeiridis et al. [24] found that BRJ significantly attenuated increases in ambulatory SBP, DBP, and MAP compared with placebo, indicating a protective effect on BP elevation during RT.

### 3.2. Short-Term BRJ Effects on BP Responses to Exercise

Four studies have evaluated the short-term effects of BRJ supplementation (3–15 days) prior to AT in clinical populations. Choi et al. (2016) [28] administered BRJ daily for 15 days (70 mL/day, ~5.6 mmol NO_3_^−^) to prehypertensive men and reported significant reductions in both resting and exercise SBP, DBP and MAP compared with placebo. Similarly, Eggebeen et al. [25] provided 1 week of daily BRJ (~6 mmol NO_3_^−^) to elderly patients with heart failure and found significant reductions in SBP at rest and during a submaximal AT protocol. In older patients with PAD, Kim et al. [29] reported that 5–6 days of BRJ supplementation (~9.7 mmol NO_3_^−^/day) significantly decreased DBP and MAP at peak exercise stages compared with placebo. By contrast, Hirai et al. [27] administered 9 days of BRJ (~13 mmol NO_3_^−^/day) to males with heart failure and observed no significant changes in resting or exercise BP.

Additionally, two studies have assessed the short-term effects of BRJ supplementation on BP during RT. Broxterman et al. [30] administered 3 days of nitrate-rich BRJ (~6.4 mmol NO_3_^−^/day) to hypertensive patients who were not taking antihypertensive medications. They observed significant reductions in SBP, DBP, and MAP during knee-extensor exercise and in DBP during handgrip exercise compared with placebo. In contrast, Kim et al. (2024) [29] provided 5–6 days of BRJ (~9.7 mmol NO_3_^−^/day) to older PAD patients but found no significant differences in BP between the conditions during standardized RT protocols.

### 3.3. Long-Term BRJ Supplementation and Exercise Training

Only one pilot study by Shaltout et al. [7] has examined the combined effects of long-term BRJ supplementation (4–6 weeks) with supervised AT on BP outcomes in patients with HTN or heart failure. While AT protocol alone significantly improved aerobic endurance and reduced resting SBP in both the BRJ and placebo groups, no additional BP-lowering effect was observed with BRJ supplementation compared to placebo. Both groups experienced similar improvements in arterial compliance and exercise tolerance, suggesting that regular AT remains a potent intervention for BP control and cardiovascular function regardless of adjunctive BRJ supplementation in these populations.

### 3.4. Summary and Interpretation of BRJ Findings

Synthesizing the evidence across acute, short-term, and long-term BRJ supplementation studies reveals several consistent patterns. Acute and short-term dosing (typically 70–500 mL/day or ~6–13 mmol NO_3_^−^ for 3–15 days, ingested ~30–180 min pre-exercise) reliably elevates plasma NO_3_^−^ and NO_2_^−^ levels and appears most effective at attenuating BP during moderate-intensity (e.g., 50% HRR, or ~60% VO_2peak_) AT in populations with mild-to-moderate vascular dysfunction (e.g., pre-HTN, PAD, obesity) and in untreated hypertensive individuals. In contrast, patients with advanced cardiovascular disease (e.g., heart failure, CAD) or those receiving antihypertensive medications show little or no BP improvement, suggesting that endothelial impairment, structural vascular changes, or pharmacotherapy may blunt the hemodynamic response to dietary nitrate. Evidence from the single long-term study [7] indicates that adding BRJ to supervised AT over four or six weeks did not provide additional benefits beyond exercise alone in hypertensive or heart failure patients, despite clear improvements in endurance and arterial compliance from the training program itself.

Taken together, these findings suggest that BRJ is most likely to provide additive or synergistic BP-lowering effects when combined with exercise in individuals with early or moderate vascular dysfunction who are not on antihypertensive therapy. The most consistent effects occur during moderate-intensity, large-muscle AT rather than high-intensity or small-muscle RT, where sympathetic activation may counteract vasodilation. Furthermore, optimal dosing protocols across studies center on 6–13 mmol nitrate per day, consumed 30–180 min before exercise.

### 3.5. Limitations of Current Evidence and Future Research Directions for BRJ

Although acute and short-term studies suggest that BRJ supplementation can lower BP during or after exercise in some clinical populations, the current evidence is limited by small sample sizes, short intervention durations, and heterogeneous patient characteristics. Future research should focus on clarifying population-specific responses to identify which groups (e.g., hypertensive, PAD, or heart failure patients) benefit most from BRJ combined with exercise. Standardization of BRJ dosing, NO_3_^−^ content, and timing relative to exercise is also needed, along with dose–response studies to determine optimal supplementation strategies. Long-term interventions (≥8–12 weeks) are particularly important to assess whether chronic BRJ intake provides additive or synergistic effects beyond exercise alone. Comparative studies across AT, RT, and combined exercise modalities, as well as investigations into the impact of exercise intensity, will further elucidate how training type moderates BP responses. Finally, inclusion of mechanistic outcomes (e.g., endothelial function, arterial stiffness, autonomic regulation, and NO biomarkers) will help clarify the physiological basis of BRJ-induced BP changes.

An additional consideration is the potential interaction between BRJ-derived nitrates and antihypertensive medications. Given that NO_3_^−^ supplementation enhances NO bioavailability and promotes vasodilation, additive BP-lowering effects may occur when combined with agents such as angiotensin-converting enzyme (ACE) inhibitors, calcium channel blockers, or nitrates [45]. Although no serious adverse interactions have been reported in the included studies, future trials should systematically monitor medication use and assess potential synergistic or hypotensive effects in medicated patients.

## 4. Green Tea

GT (*Camellia sinensis*) is a widely consumed herbal supplement rich in polyphenols such as epigallocatechin gallate (EGCG), epigallocatechin, epicatechin-3 gallate, epicatechin, gallocatechin, and gallocatechin gallate [35,46,47]. Owing to these bioactive compounds, particularly EGCG, GT has attracted attention for its potential health benefits [47]. Evidence suggests that GT supplementation may contribute to modest reductions in SBP and DBP [46], largely through its antioxidant and anti-inflammatory properties [35,46].

To date, six studies have examined the effects of GT supplementation on exercise-induced BP responses and adaptations. These include one acute study [31], one short-term intervention [32], and four long-term trials [4,33,34,35]. While the acute (single dose, ~30 min before exercise) and short-term studies (up to three weeks of supplementation) assessed BP responses after exercise, the majority of available research has focused on longer-term interventions (6–9 weeks), investigating the combined effects of GT supplementation with structured exercise training on resting or ambulatory BP outcomes in various clinical populations.

### 4.1. Acute and Short-Term GT Effects on BP Responses to Exercise

Research on GT supplementation and exercise-related BP responses is limited, with only one acute and one short-term study available. Neto et al. [31] tested a single GT dose (~2 g, 636 mg EGCG, ~20 mg caffeine) before AT in hypertensive adults. Although both GT and placebo sessions induced post-exercise hypotension (PEH), GT blunted the systolic response and induced a hypertensive diastolic reaction, suggesting acute GT ingestion may attenuate BP benefits of AT.

In contrast, Arazi et al. [32] evaluated the effects of GT extract consumption (~75 mg EGCG twice daily) for three weeks on BP responses to a single RT session in hypertensive women. Both RT and GT groups reduced SBP and DBP post-exercise, with only modest additional effects of GT on MAP and rate-pressure product (RPP). Overall, GT did not enhance BP reductions beyond exercise alone in the short term.

### 4.2. Long-Term GT Supplementation and Exercise Training

Four long-term trials have evaluated the combined effects of GT and exercise training. In overweight/obese adults, both Touli et al [33] and Amozadeh et al. [34] found that GT combined with AT reduced SBP and DBP compared to control, but without added benefit beyond training alone. Similarly, Golpasandi et al. [35] reported BP reductions with GT (800 mg daily, ∼320 mg EGCG), HIIT, and their combination for eight weeks in obese men with T2DM, but no interaction effect. By contrast, Taati et al. [4] showed that GT extract (500 mg daily, ~150 mg EGCG) with low-intensity RT (i.e., 50% 1RM) in hypertensive women yielded greater improvements in office and 24 h BP, MAP, and RPP compared to either intervention alone, suggesting possible synergy with resistance-based protocols.

### 4.3. Summary and Interpretation of GT Findings

Research on GT supplementation and exercise reveals that its effects on BP are highly dependent on patient characteristics, exercise modality, and supplementation duration. Acute and short-term studies showed mixed outcomes. In physically active hypertensive patients, acute ingestion of GT containing ~20 mg caffeine attenuated systolic PEH and induced a transient diastolic hypertensive response during AT [31], suggesting that caffeine content and its vasoconstrictive properties may offset GT’s expected BP-lowering effects. Conversely, a three-week GT regimen (~75 mg EGCG/day) modestly improved post-exercise MAP and RPP responses to RT in hypertensive women, although effects on SBP and DBP were limited [32].

Long-term studies (i.e., 6–9 weeks) demonstrated more consistent BP reductions, primarily driven by exercise training, with GT showing variable additive effects across populations. In overweight and obese men and women, AT alone reduced BP, and GT provided no additional benefit when combined with training [33,34]. In obese men with T2DM, both GT alone and HIIT alone lowered BP, but no synergistic interaction was observed [35]. In contrast, among women with HTN, GT supplementation combined with RT produced greater improvements in resting and ambulatory BP, MAP, and RPP than either intervention alone, highlighting a potential synergistic effect in this higher-risk group [4].

Taken together, the evidence suggests that GT supplementation may be most beneficial in patients with established HTN, particularly when combined with low-intensity RT (e.g., 50% 1RM), whereas in overweight, obese, or diabetic populations, exercise remains the dominant driver of BP reduction, with GT acting as either a modest independent intervention or offering no additional advantage.

### 4.4. Limitations of Current Evidence and Future Research Directions for GT

The current body of evidence on GT supplementation and exercise-induced BP responses is limited, with only a single acute and short-term trial available. Most evidence comes from long-term interventions, but even here, findings are inconsistent and often confounded by the strong BP-lowering effects of exercise itself, making it difficult to isolate GT’s contribution.

Additionally, study populations have been heterogeneous (i.e., hypertensives, obese individuals, patients with T2DM), and supplementation protocols varied widely in form, dose, duration, and caffeine content. The influence of caffeine, present in some preparations, remains unclear and may attenuate BP-lowering effects. Accordingly, future studies should use standardized GT formulations, evaluate caffeine-free options, and apply rigorous designs across diverse patient groups. Trials with ambulatory BP monitoring, mechanistic biomarkers, and RT interventions in hypertensives appear especially promising.

Potential interactions between GT supplementation and antihypertensive therapy should also be considered. Catechins may influence endothelial function and the renin–angiotensin–aldosterone system (RAAS) activity, while caffeine-containing preparations could transiently elevate BP or interact with beta-blockers [48]. Furthermore, polyphenols may affect drug-metabolizing enzymes, potentially altering pharmacokinetics of certain medications [49]. Future studies should carefully control for medication regimens and distinguish between caffeinated and decaffeinated formulations.

## 5. Curcumin

CN, the principal natural compound in turmeric (*Curcuma longa*), is a yellowish polyphenol with diverse therapeutic properties linked to its chemical structure, which allows interactions with cellular molecules and mediates antioxidant and anti-inflammatory effects [50,51,52]. In recent years, CN has gained global research attention [53], with studies highlighting its anti-inflammatory actions [52,53,54] and its potential in the prevention and treatment of cardiovascular disease (CVD) [12,55] and other chronic disorders [13,53].

To date, four studies have investigated the combined effects of CN supplementation and exercise training on BP adaptations. All studies were long-term interventions (i.e., 6–16 weeks), with no trials exploring acute or short-term effects of CN on exercise-related BP responses. Three studies examined AT or HIIT in overweight, obese, or T2DM individuals [16,36,37], while one study assessed the interaction of CN with RT in older adults at risk for MetS [6].

### 5.1. Long-Term CN Supplementation and Exercise Training

Osali et al. [36] examined six weeks of moderate-intensity AT (i.e., 65–75% HRR) combined with nano-CN supplementation (80 mg/day) in older women (60–65 years) with MetS. Both AT and CN alone reduced SBP, but the combined intervention led to greater reductions compared with control. Similarly, Darmian et al. [37] reported that eight weeks of AT (walking/jogging at 60–75% HR_max_) plus turmeric supplementation (∼23 mg CN/day) in middle-aged women with T2DM reduced both SBP and DBP more effectively than either AT or CN alone.

In contrast, Dabidi Roshan et al. [16] found no additive effects for the combined interventions. They investigated eight weeks of low- and moderate-volume HIIT with or without nano-CN supplementation (80 mg/day) in obese menopausal women. SBP reductions were observed only with CN alone, while both CN and HIIT reduced DBP.

In the only study conducted on RT, Juesas et al. [6] compared 16 weeks of high-RT (accentuated eccentric or maximum strength protocols) with or without CN supplementation (500 mg/day) in older adults at risk of MetS. All RT groups significantly reduced SBP and DBP compared to controls. Notably, CN supplementation alone prevented the BP increases observed in placebo controls, and the RT + CN group achieved additional reductions in DBP compared to placebo.

### 5.2. Summary and Interpretation of CN Findings

Evidence on CN supplementation with exercise training is limited to four long-term trials, but collectively they suggest favorable effects on BP regulation in at-risk populations. Across studies, CN was administered in doses ranging from ~23 mg/day to 500 mg/day over 6–16 weeks, combined with AT, HIIT, or RT.

Patterns emerge when findings are considered by participant characteristics. In older women with MetS [36] and women with T2DM [37], CN enhanced the BP-lowering effects of moderate-intensity AT (e.g., 60–75% HR_max_ or HRR), suggesting potential synergy between CN and aerobic modalities in populations with impaired metabolic and vascular health. In obese menopausal women [16], CN and HIIT each improved DBP, but their combination did not yield additive benefits, which may reflect physiological limits in vascular adaptation or insufficient dosing relative to the training intensity. In older adults at risk of MetS [6], CN combined with high-RT protocols amplified DBP improvements beyond training alone and helped blunt the rise in BP observed in placebo controls, pointing toward protective or stabilizing effects in aging populations.

Taken together, the available evidence indicates that CN supplementation, particularly in metabolically compromised or older individuals, may complement AT and RT to improve BP regulation. However, the magnitude and consistency of effects appear influenced by exercise modality, CN formulation/dose, and baseline health condition.

### 5.3. Limitations of Current Evidence and Future Research Directions for CN

Current evidence on CN with exercise is limited by small sample sizes, short intervention periods (i.e., 6–16 weeks), and a lack of acute or short-term studies. Most trials focused on older adults or patients with metabolic disturbances, restricting generalizability. Heterogeneity in formulations (i.e., turmeric, nano-CN, standardized extracts) and doses (i.e., 23–500 mg/day) also complicates comparisons, while mechanistic outcomes were rarely assessed. Future studies should test standardized CN preparations at varied doses, explore both acute and chronic effects across different exercise modalities, and include mechanistic biomarkers. Larger, longer trials in diverse populations are needed to clarify CN’s role in exercise-related BP regulation.

CN may also interact with antihypertensive medications due to its anti-inflammatory and vasodilatory properties, potentially enhancing BP-lowering effects. Additionally, CN has been reported to influence cytochrome P450 activity and drug metabolism, which may alter plasma levels of certain cardiovascular drugs [56]. Careful monitoring of medicated patients and pharmacodynamic assessments are warranted in future trials.

## 6. Resveratrol

RSV (3,5,4′-trihydroxystilbene) is a naturally occurring polyphenolic compound belonging to the stilbene family, found in more than 70 plant species. Its main dietary sources include grapes, peanuts, cacao, and berries [11,57]. Numerous studies have demonstrated the antihypertensive effects of RSV in various preclinical models of HTN, primarily through its antioxidant capacity, stimulation of endothelial NO production, inhibition of vascular inflammation, and prevention of platelet aggregation [11,58,59].

To date, only three studies have investigated the effects of RSV supplementation on BP responses to exercise (one acute and two long-term interventions) mainly in populations with cardiovascular or metabolic disorders.

### 6.1. Acute RSV Effects on BP Responses to Exercise

The only acute study, conducted by Gonzaga et al. [38], examined the isolated and combined effects of a single 500 mg dose of trans-RSV and BRJ extract on post-exercise BP recovery in men with CVD. Despite increases in plasma NO_3_^−^ and NO-related metabolites, no significant differences in SBP, DBP, or MAP were observed between RSV, BRJ, RSV + BRJ, or placebo conditions. These results indicate that a single RSV dose may not acutely influence BP responses during or after moderate-intensity AT (i.e., 60% HRR), at least in individuals with established CVD.

### 6.2. Long-Term RSV Supplementation and Exercise Training

Long-term studies have provided more mixed findings. Dorani and Hosseini [17] reported that eight weeks of AT combined with 400 mg/day RSV supplementation significantly reduced SBP and DBP in overweight women, though the reductions were comparable to those achieved by AT or RSV alone, suggesting no additive effect. Conversely, Nicolau et al. [39] found that 60 days of RSV intake (300 mg/day) without structured exercise increased BP in sedentary older women, while active women maintained stable BP levels. This suggests that exercise training may mitigate potential adverse BP responses to RSV in older adults.

### 6.3. Summary and Interpretation of RSV Findings

Collectively, evidence from the limited studies suggests that RSV supplementation alone exerts inconsistent effects on BP, which appear to depend strongly on participants’ health status and physical activity level. Sedentary or older individuals may experience neutral or even adverse BP responses, whereas combining RSV with regular exercise, particularly AT, tends to support BP reduction or maintenance within normal ranges. However, the absence of consistent synergistic effects between RSV and exercise indicates that the hemodynamic benefits of RSV may be secondary to, or dependent on, exercise-induced vascular adaptations.

### 6.4. Limitations of Current Evidence and Future Research Directions for RSV

Current evidence on RSV and exercise-related BP adaptations is limited by small sample sizes, short intervention durations (i.e., 6–8 weeks), and heterogeneous doses (i.e., 300–500 mg/day). Moreover, none of the studies investigated the effects of RSV combined with RT or interval training, which could elicit different vascular responses. Future studies should focus on dose–response relationships, the interaction between RSV bioavailability and exercise intensity, and sex- and age-specific differences in BP regulation. Long-term, well-controlled randomized trials using standardized RSV preparations are needed to clarify whether RSV offers meaningful cardiovascular benefits beyond those of exercise training alone.

RSV’s effects on AMP-activated protein kinase (AMPK)/Sirtuin 1 (SIRT1) signaling, endothelial function, and vascular tone suggest the possibility of additive interactions with antihypertensive therapies [60]. Moreover, RSV may influence platelet aggregation and drug-metabolizing pathways [61], which could be clinically relevant in patients receiving combination cardiovascular therapy. Future investigations should evaluate safety profiles and potential pharmacodynamic interactions in medicated populations.

## 7. Garlic

Garlic (*Allium sativum*), the edible bulb of a plant from the lily family, is widely recognized for both its culinary value and traditional medicinal properties. Its cardiovascular benefits are primarily attributed to bioactive sulfur-containing compounds such as allicin, *S*-allyl cysteine (SAC), and diallyl disulfide, which exert antihypertensive, antioxidant, and anti-inflammatory effects [9,62,63]. A meta-analysis of 12 clinical trials involving 553 hypertensive participants confirmed that garlic supplementation significantly reduces SBP and DBP [14]. More recent randomized controlled trials further support its antihypertensive potential in medicated Grade I hypertensive patients receiving low-dose SAC from aged garlic extract [64] and in prehypertensive individuals supplemented with freeze-dried garlic extract, where significant improvements in BP, lipid profile, and NO levels were observed [65]. Proposed mechanisms include enhanced NO bioavailability, modulation of ACE activity, improved endothelial responsiveness, and antioxidant effects. Despite the growing body of evidence supporting garlic as a standalone intervention, only three studies to date have examined its effects in conjunction with structured exercise training on BP adaptations.

### 7.1. Long-Term Garlic Supplementation and Exercise Training

Towhidi et al. [40] examined the effects of eight weeks of AT and garlic extract (6 g/day) in obese hypertensive men (mean age 53 ± 7.6 years). Both AT and garlic supplementation significantly reduced SBP and DBP compared with controls, with no additional benefit from combining the two interventions. Similarly, Kaleh et al. [41] evaluated the effects of eight weeks of progressive AT (55–65% HR reserve, 30–55 min/session, 3 sessions/week) and garlic supplementation (1000 mg twice daily) in postmenopausal obese women with HTN. SBP significantly decreased in all intervention groups (AT, garlic, AT + garlic) relative to controls, while DBP was significantly reduced only in the combined AT + garlic group.

More recently, Ried et al. [42] conducted a 12-week randomized controlled trial in middle-aged recreational endurance athletes with elevated arterial stiffness, investigating the effects of Kyolic aged garlic extract (2.4 or 9.6 g/day) while participants maintained their habitual moderate-to-high intensity endurance training. Although garlic supplementation significantly improved pulse wave velocity, aerobic fitness, lactate threshold, recovery indices, and cardiovascular proteomic biomarkers, no significant differences in resting SBP or DBP were observed between the garlic and placebo groups after 12 weeks. These findings suggest that in already trained individuals with preserved BP control, vascular and performance adaptations may occur independently of resting BP changes.

### 7.2. Summary and Interpretation of Garlic Findings

The limited available evidence indicates that garlic supplementation improves BP regulation in hypertensive and obese individuals, whether used alone or in conjunction with AT. The antihypertensive effects appear consistent across studies, but the presence or absence of synergistic effects may depend on participants’ sex, hormonal status, or differences in garlic dosage and formulation. While both men and women benefited from BP reductions, the greater diastolic improvement observed in postmenopausal women [41] may reflect enhanced vascular responsiveness or oxidative stress modulation in this group. Collectively, current evidence suggests that garlic supplementation may contribute to BP reductions in hypertensive and obese populations, particularly when combined with AT. However, in normotensive or well-trained individuals with controlled baseline BP, improvements may manifest primarily through enhanced vascular function and aerobic capacity rather than additional reductions in resting BP.

### 7.3. Limitations of Current Evidence and Future Research Directions for Garlic

Current evidence on the combined effects of garlic supplementation and exercise training on BP regulation remains limited to a small number of randomized controlled trials with heterogeneous designs and populations. While BP reductions have been observed in hypertensive and obese individuals, findings in physically active or normotensive cohorts suggest that improvements may primarily involve arterial stiffness or aerobic performance rather than additional reductions in resting BP. Variability in garlic formulation, dosage, intervention duration, and exercise protocols further limits comparability across studies. Future adequately powered trials using standardized preparations, clearly defined exercise prescriptions, and comprehensive cardiovascular endpoints are needed to determine whether garlic provides additive or synergistic benefits beyond exercise alone.

Garlic supplementation may exert additive BP-lowering effects when combined with antihypertensive agents, particularly ACE inhibitors or diuretics, due to its influence on NO and hydrogen sulfide (H_2_S) pathways [63]. Additionally, garlic’s mild antithrombotic properties may interact with anticoagulant or antiplatelet therapies [66]. Future studies should stratify participants by medication use and monitor potential synergistic or excessive hypotensive responses.

## 8. Mechanistic Overview of Plant-Derived Bioactives and Exercise in BP Regulation

**Figure 1:**
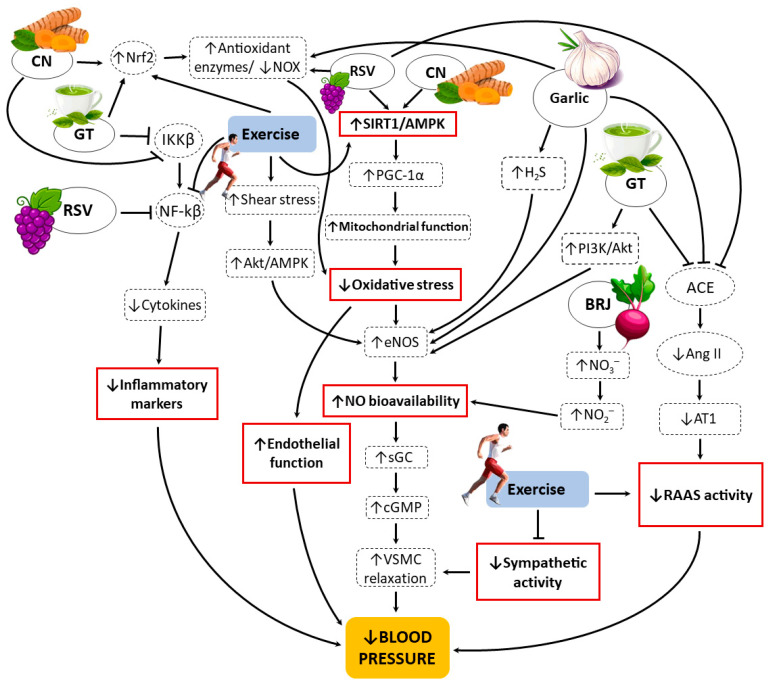
Proposed integrated mechanistic pathways through which beetroot juice (BRJ), curcumin (CN), green tea (GT), resveratrol (RSV), garlic, and exercise training regulate blood pressure. The figure summarizes the main converging mechanisms (the solid boxes) leading to endothelial improvement, vascular relaxation, and decreased blood pressure. These represent the predominant proposed pathways, although additional signaling routes may also contribute to the antihypertensive effects of each agent. ↑ increase, ↓ decrease, → activation, ⊣ inhibition, Nrf2: Nuclear factor erythroid 2 like factor 2, IKKβ: IκB kinase beta, NF-kβ: nuclear factor-kappa beta, NOX: NADPH oxidase, AMPK: AMP-activated protein kinase, PGC-1α: peroxisome proliferator-activated receptor gamma coactivator 1-alpha, eNOS: endothelial nitric oxide synthase, NO: nitric oxide, sGC: soluble guanylate cyclase, cGMP: cyclic guanosine monophosphate, VSMC: vascular smooth muscle cells, H_2_S: hydrogen sulfide, PI3K: phosphatidylinositol-3 kinase, NO_3_^−^: nitrate, NO_2_^−^: nitrite, ACE: angiotensin converting enzyme, Ang II: angiotensin II, AT1: angiotensin II type 1, RAAS: renin–angiotensin–aldosterone system.

Plant-derived bioactives and exercise training converge on a set of vascular-regulatory hubs that together lower systemic BP (Figure 1). A primary shared axis is the NO signaling cascade: dietary NO_3_^−^ from BRJ is reduced via the enterosalivary pathway and tissue NO_2_^−^ reduction to increase NO bioavailability and stimulate soluble guanylate cyclase (sGC), causing vascular smooth muscle cell (VSMC) relaxation and reduced peripheral resistance [67,68]. Several phytochemicals (e.g., CN, GT catechins, RSV, and garlic) augment endothelial NO by upregulating endothelial nitric oxide synthase (eNOS) via phosphatidylinositol-3 kinase (PI3K)/Akt or AMPK)/SIRT1 signaling, preserving eNOS coupling through antioxidant/Nrf2-driven induction of antioxidant enzymes (e.g., superoxide dismutase, catalase, glutathione peroxidase) and tetrahydrobiopterin (BH_4_) preservation, or by complementary gasotransmitter signaling (garlic → H_2_S) that synergizes with NO [60,63,69,70,71,72]. These NO-centered actions are reinforced by reduced oxidative stress and lower inflammatory signaling, which together protect endothelial function and maintain vasodilatory responsiveness.

Beyond NO, members of this group exert complementary effects on mitochondrial and neurohormonal control of vascular tone. RSV and exercise strongly activate SIRT1/AMPK → peroxisome proliferator-activated receptor gamma coactivator 1-alpha (PGC-1α) to improve mitochondrial biogenesis and reduce mitochondrial ROS, indirectly sustaining NO signaling and endothelial health [73,74]. Garlic, RSV and some catechins additionally attenuate RAAS signaling, reducing vasoconstrictive drive and salt-retaining hormonal effects [48,63,75], while regular exercise lowers sympathetic outflow and RAAS activity and increases endothelial shear stress that directly stimulates eNOS [76,77]. Together, these overlapping mechanisms (i.e., increased NO bioavailability, reduced oxidative stress and inflammation, lower RAAS/sympathetic drive, and improved SIRT1/AMPK–mitochondrial function) collectively explain how the plant-derived bioactives and exercise synergistically improve endothelial function, reduce vascular resistance, and lower BP in clinical and experimental studies.

## 9. Limitations

Several limitations of the current evidence should be acknowledged. Although participant characteristics including sex distribution, age range, and clinical condition are summarized in Table 1, none of the included studies were designed or powered to evaluate sex- or age-specific differences in BP responses. Therefore, potential moderating effects of biological sex, hormonal status, or aging on supplementation–exercise interactions remain unclear.

In addition, while some trials included participants receiving stable antihypertensive therapy, reporting of medication regimens and polypharmacy was inconsistent. In several studies, medication use was either not specified or temporarily withheld prior to testing in acute protocols [18], limiting conclusions regarding real-world pharmacological interactions.

Finally, comorbidity profiles varied substantially across studies (e.g., obesity, PAD, COPD, established HTN, physically active individuals), which may partly explain heterogeneity in BP responses and restrict generalizability to patients with complex cardiovascular risk profiles.

## 10. Conclusions and Practical Implications

The available evidence demonstrates that plant-derived bioactives and exercise training exert complementary and mechanistically convergent effects on BP regulation. Among the botanicals reviewed, BRJ shows the most consistent benefits through enhanced NO bioavailability via the NO_3_^−^–NO_2_^−^–NO pathway, leading to improved endothelial function, vascular relaxation, and lower peripheral resistance. CN and GT catechins primarily act through potent antioxidant and anti-inflammatory mechanisms: upregulating Nrf2-dependent antioxidant enzymes, suppressing nuclear factor-kappa beta (NF-κB) signaling, and supporting endothelial NO synthesis. Garlic offers dual benefits by stimulating endothelial NO and H_2_S production while inhibiting ACE activity, thereby mitigating vasoconstriction and fluid retention. RSV acts as a metabolic regulator by activating SIRT1/AMPK–PGC-1α signaling, improving mitochondrial efficiency, and maintaining redox and endothelial homeostasis. Exercise training, in turn, may reinforce these effects by promoting shear stress–mediated eNOS activation, reducing sympathetic and RAAS activity, and further enhancing antioxidant capacity and endothelial health.

From a translational standpoint, integrating exercise with targeted plant-based supplementation represents a physiologically grounded and low-risk strategy for improving BP control, particularly in individuals with early or moderate vascular dysfunction. The magnitude and consistency of BP responses depend on factors such as supplement type, dose, timing relative to exercise, duration of intervention, and baseline health status. Personalized combinations that leverage complementary mechanisms (e.g., BRJ for NO support, CN or GT for redox balance, RSV for mitochondrial optimization, and garlic for RAAS modulation) hold promise for synergistic BP reduction, though this hypothesis requires confirmation in well-controlled clinical trials. Future research should prioritize standardized protocols, mechanistic biomarkers, and long-term outcomes to establish evidence-based recommendations for clinical implementation.

## Figures and Tables

**Table 1:** Characteristics of the studies included.

Author, Year	Population (*n*), Age	Medication	Study Design	Dose & Duration	Adverse Drug Reactions	Exercise Protocol	Main Outcomes
Beetroot juice (BRJ)							
Kenjale et al., 2011 [18]	Males and females with PAD (*n* = 8), 67 ± 13 years	On antihypertensive medication (with ≥12 h washout)	Double-blind randomized crossover	500 mL BRJ (~6.2 mmol NO_3_^−^), 180 min before exercise	NR	Gardner treadmill test (walking, 2 mph with a 2%-grade increase every 2 min to exhaustion)	↑ plasma NO_3_^−^ and NO_2_^−^ with BRJ ↓ DBP at rest/exercise with BRJ No difference in SBP at rest/exercise
Berry et al., 2015 [19]	Males and females with COPD (*n* = 15), 69.6 ± 8.5 years	On medication (unspecified)	Single-blind randomized crossover	140 mL BRJ (~7.6 mmol NO_3_^−^), 150 min before exercise	NR	Cycling on an ergometer (75% of MPO to exhaustion)	↑ plasma NO_3_^−^ and NO_2_^−^, and ↓ resting SBP and ↓ DBP post-exercise with BRJ
Amaral et al., 2019 [20]	Postmenopausal women with HTN (*n* = 13), 50–70 years	On antihypertensive medication (unspecified)	Double-blind randomized crossover	350 mL BRJ (~7.3 mmol NO_3_^−^) or PL (~1.3 mmol NO_3_^−^), ~120 min before exercise	NR	Running on a treadmill (65–70% of HRR, 40 min)	↑ Salivary NO_2_^−^ with BRJ ↔ SBP and DBP during 90 min post-exercise recovery
Bezerra et al., 2019 [21]	Obese men (*n* = 14), 20–30 years	NR	Double-blind randomized crossover	200 mL BRJ (~12.9 mmol NO_3_^−^), 60 min before exercise	NR	Running on a treadmill (50% of HRR, 40 min)	↑ plasma NO_3_^−^ and NO_2_^−^, ↓ ambulatory SBP with BRJ ↔ DBP with BRJ
van der Avoort et al., 2021 [22]	Males and females with PAD (*n* = 18), 73 ± 8 years	On antihypertensive medication (unspecified)	Double-blind randomized crossover	70 mL BRJ (∼6.5 mmol NO_3_^−^), 180 min before exercise	No	Running on a treadmill (3.2 km/h with a 2%-grade increase every 2 min to exhaustion)	↑ plasma NO_3_^−^ and NO_2_^−^ with BRJ ↔ SBP, DBP, and MAP with BRJ
Gonzaga et al., 2025 [23]	Males with CAD (*n* = 14), 41–72 years	Calcium channel blocker, ACE inhibitors, ARB	Triple-blind randomized crossover	500 mg BRJ (50 mg or ∼0.81 mmol NO_3_^−^), 30 min before exercise	NR	Treadmill running (60% of HRR, 30 min)	↑ SBP at the first min of recovery in all protocols ↔ BP responses with BRJ
Zafeiridis et al., 2019 [24]	Males and females with HTN (*n* = 18), 44 ± 2.6 years	Not on medication	Double-blind randomized crossover	500 mg BRJ (∼8.1 mmol NO_3_^−^), 150 min before exercise	No	Isometric HG using a digital dynamometer (2 min, 30% of MVC)	↑ plasma NO_2_^−^, and ↓ resting SBP, DBP, and MAP with BRJ ↓ ambulatory SBP, DBP, and MAP rise during recovery
Eggebeen et al., 2016 [25]	Males and females with HFpEF (*n* = 20), 69 ± 7 years	ACE inhibitors, Diuretics, Loop diuretics, Beta-blockers, Calcium channel blockers, ARB	Double-blind randomized crossover for acute phase + single-blind all-treated for short-term phase	70 mL BRJ (∼6.1 mmol NO_3_^−^), ∼ 90–120 min before exercise (acute), 6–8 days (short-term)	No	Cycling on an ergometer (75% of MPO to exhaustion)	↑ plasma NO_3_^−^ and NO_2_^−^ with acute and short-term BRJ Acute: ↓ resting SBP Short-term: ↓ SBP at rest and exercise
Benjamim et al., 2024 [26]	Postmenopausal women with HTN (*n* = 14), 59 ± 4 years	ACE inhibitors, Diuretics, ARB	Triple-blind randomized crossover for acute and short-term phases	Acute: 140 mL BRJ (∼12.8 mmol NO_3_^−^), 150 min before exercise Short-term: 70 mL BRJ (∼6.4 mmol NO_3_^−^) for 6 days	No	Bruce’s modified test on a treadmill (1.7 mph, 0% grade, gradually increase every 2 min to exhaustion)	↑ plasma NO_3_^−^ and NO_2_^−^ with acute and short-term BRJ Acute: ↓ SBP and MAP at 50 min post-exercise
Hirai et al., 2017 [27]	Males with HFrEF (*n* = 10), 63 ± 5 years	ACE inhibitors, Diuretics, Beta-blockers, Calcium channel blockers, ARB	Double-blind randomized crossover	140 mL BRJ (∼12.9 mmol NO_3_^−^) for 9 days	No	Cycling on an ergometer with two intensities (low and high) to exhaustion	↑ plasma NO_3_^−^ with BRJ No difference in BP responses to low- and high-intensity AT between the conditions
Choi et al., 2016 [28]	Prehypertensive men (*n* = 11), 23 ± 1 years	Not on antihypertensive medication	Double-blind randomized crossover	70 mL BRJ (∼5.6 mmol NO_3_^−^) for 15 days	NR	Cycling on an ergometer with low (30% VO_2peak_) and high (60% VO_2peak_) intensity to exhaustion	↑ plasma NO_3_^−^/NO_2_^−^ with BRJ ↓ resting and exercise (both intensities) SBP, DBP, and MAP with BRJ
Kim et al., 2024 [29]	Males and females with PAD (*n* = 11), 52–80 years	On antihypertensive medication (unspecified)	Double-blind randomized crossover	140 mL BRJ (∼9.7 mmol NO_3_^−^) for 4–6 days	No	AT (day 5 or 6): Gardner treadmill test (walking, 2 mph with a 2%-grade increase every 2 min to exhaustion) RT (day 4): plantar flexion (20 contractions/min) and HG (40% MVC) tests to fatigue	↑ plasma NO_3_^−^ and NO_2_^−^ with BRJ Lower peak DBP and MAP during AT with BRJ No difference in BP responses between the conditions during RT
Broxterman et al., 2022 [30]	Males and females with HTN (*n* = 26), 18–65 years	ACE inhibitors or ARB, Diuretics, Beta-blockers, Calcium channel blockers	Double-blind randomized crossover with two parallel arms: being drug-naïve (Off-Meds) or not (On-Meds)	70 mL BRJ (∼6.4 mmol NO_3_^−^) for 3 days	NR	Intermittent isometric HG (15, 30, and 45% MVC) and dynamic KE (40, 60, and 80% P_peak_) exercise for 3 min, and 2 min rest	↑ plasma NO_2_^−^ with BRJ in both arms ↓ resting SBP, DBP, and MAP with Off-Meds BRJ vs. On-Meds BRJ ↓ DBP during HG exercise in Off-Meds BRJ vs. PLA ↓ SBP, DBP, and MAP during KE exercise with BRJ
Shaltout et al., 2017 [7]	Males and females with HTN (*n* = 26, 65 ± 5 years) or with HFpEF (*n* = 20, 69 ± 7 years)	ACE inhibitors, Diuretics, Loop diuretics, Beta-blockers, Calcium channel blockers, ARB	Double-blind randomized crossover with two parallel arms for HTN and HFpEF patients: BRJ and PLA	70 mL BRJ containing ∼8 mmol NO_3_^−^ (every day, HTN study) or ∼6.1 mmol NO_3_^−^ (3 days/week, HFpEF study) for 4 weeks	No	HTN: walking (RPE 12–13, 50 min, 3 sessions/week, 6 weeks) HFpEF: cycling (40–60% MPO, 20 min) and walking (50–70% HRR, 20 min), 3 sessions/week for 4 weeks	HTN: ↑ plasma NO_3_^−^ and NO_2_^−^ with BRJ HFpEF: ↑ plasma NO_3_^−^ with BRJ HTN and HFpEF: ↓ resting SBP in both groups No additive BRJ effect
Green tea (GT)							
Neto et al., 2017 [31]	Males and females with HTN (*n* = 15), 53 ± 3 years	On antihypertensive medication (unspecified)	Double-blind randomized crossover	2 g of GT (636 mg EGCG, 20 mg caffeine), 30 min before exercise	NR	Treadmill walking (60–85% HR_max_, 60 min)	Systolic and diastolic PEH with PLA in the most recovery time points A significant PEH inhibition with GT
Arazi et al., 2014 [32]	Females with HTN (*n* = 24), 46 ± 6 years	ACE inhibitors, Beta-blockers, Calcium channel blockers	Double-blind randomized with three parallel arms: (1) RT + GT, (2) RT + PLA, and (3) PLA	500 mg of GT extract (∼150 mg EGCG, 50 mg caffeine) daily for 3 weeks	No	Acute low-intensity RT (50% 1RM, 2 circuits of 10 reps, 6 exercises)	↓ SBP and DBP 60 min post-exercise and MAP 15–60 min post-exercise in both RT + GT and RT + PLA groups
Touli et al., 2022 [33]	Overweight men (*n* = 40), 28–35 years	NR	Randomized with four parallel arms: (1) AT + GT, (2) AT, (3) GT, and (4) control	3 g of dry GT leaves in 200 mL hot water, brewed for 6–8 min, 3 times/day for 6 weeks	NR	AT (3 sessions/week, 45 min, 50–70% HR_max_) for 6 weeks	↓ resting SBP and DBP in both AT + GT and AT groups vs. control group
Amozadeh et al., 2018 [34]	Overweight and obese females (*n* = 39), 28 ± 6 years	NR	Randomized with three parallel arms: (1) AT + GT, (2) AT, and (3) control	∼100 mg of GT daily for 8 weeks	NR	AT (3 sessions/week, 80–90 min, 40–80% HRR) for 8 weeks	↓ resting SBP and DBP in both AT + GT and AT groups vs. control group
Golpasandi et al., 2024 [35]	Obese men with T2DM (*n* = 32), 40–55 years	NR	Double-blind randomized with four parallel arms: (1) HIIT + GT, (2) HIIT + PLA, (3) GT, and (4) control	800 mg of GT extract (∼320 mg EGCG) daily for 8 weeks	NR	HIIT (3 sessions/week, 40 min, six 1 min bouts at 90–95% HR_max_, interspersed with six 4 min rest intervals at 70–75% HR_max_) for 8 weeks	↓ resting SBP and DBP in HIIT + GT, HIIT + PLA, and GT groups
Taati et al., 2021 [4]	Females with HTN (*n* = 44), 35–55 years	ACE inhibitors, Beta-blockers, Calcium channel blockers	Double-blind randomized with four parallel arms: (1) RT + GT, (2) RT + PLA, (3) GT, and (4) control	500 mg of GT extract (∼150 mg EGCG, 50 mg caffeine) daily for 9 weeks	No	Low-intensity RT (2 sessions/week, 50% 1RM, 2 circuits of 10 reps, 6 exercises) for 6 weeks	↓ resting and 24 h SBP in RT + PLA vs. control group ↓ resting and 24 h SBP, and resting MAP in RT + GT vs. control group
Curcumin (CN)							
Osali, 2020 [36]	Females with MetS (*n* = 44), 62 ± 1 years	NR	Double-blind randomized with four parallel arms: (1) AT + CN, (2) AT + PLA, (3) CN, and (4) control	80 mg of nano-CN daily for 6 weeks	NR	Treadmill walking/running (65–75% HRR, 3 × 12–17 min) for 6 weeks	↓ resting SBP in AT + CN, AT + PLA, and CN groups vs. baseline ↓ resting SBP in AT + CN vs. control group
Darmian et al., (2022) [37]	Females with hyperlipidemic T2DM (*n* = 42), 45–60 years	NR	Single-blind randomized with four parallel arms: (1) AT + CN, (2) AT + PLA, (3) CN, and (4) control	2100 mg of turmeric powder (∼23 mg CN) daily for 8 weeks	NR	AT (3 sessions/week, 20–40 min, 60–75% HR_max_) for 8 weeks	↓ resting SBP and DBP in all groups vs. control ↓ resting SBP and DBP in AT + CN group vs. AT + PLA and CN groups ↓ resting SBP and DBP in AT + PLA group vs. CN group
Dabidi Roshan et al., (2024) [16]	Obese females (*n* = 53), 45–60 years	NR	Double-blind randomized with six parallel arms: (1) LV-HIIT + CN, (2) LV-HIIT + PLA, (3) MV-HIIT + CN, (4) MV-HIIT + PLA, (5) CN, and (6) PLA	80 mg of nano-CN daily for 8 weeks	NR	HIIT (2 sessions/week, 2–4 × 8 bouts/session) for 8 weeks The work/rest ratio was gradually increased from 1/1 to 3/1 for both LV-HIIT (20–30 s of work) and MV-HIIT (15–25 s of work)	↓ resting SBP and DBP in CN group vs. baseline ↓ resting DBP in LV-HIIT, MV-HIIT and CN groups vs. baseline
Juesas et al., (2025) [6]	Older males and females at risk of MetS (*n* = 81), 68 ± 5 years	NR	Double-blind randomized with six parallel arms: (1) Aecc + CN, (2) Aecc + PLA, (3) Max RT + CN, (4) Max RT + PLA, (5) CN, and (6) PLA	500 mg of CN extract (∼10.5 mg CN) daily for 16 weeks	No	Full-body RT (4 sets of 6 submaximal reps, 4 multi-joint exercises) performed as Aecc or Max, for 16 weeks	↓ resting SBP in all training groups vs. CN and PLA groups ↓ resting DBP in Max-Cur group vs. CN and PLA groups ↑ resting SBP and DBP in PLA, but not in CN group
Resveratrol (RSV)							
Gonzaga et al., 2024 [38]	Males with CAD (*n* = 13), 61 ± 9 years	ACE inhibitors, Beta-blockers, Calcium channel blockers, ARB	Triple-blind randomized crossover	500 mg trans-RSV, 30 min before exercise	NR	Treadmill running (60% of HRR, 30 min)	↑ SBP and MAP at the first and third min of recovery in all protocols ↔ BP responses with RSV
Dorani and Hosseini, 2021 [17]	Females with MetS (*n* = 32), 30–45 years	NR	Randomized with four parallel arms: (1) AT + RSV, (2) AT, (3) RSV, and (4) control	400 mg of RSV daily for 8 weeks	NR	Treadmill running (3 sessions/week, 60–75% HR_max_, 60 min) for 8 weeks	↓ resting SBP and DBP in all groups vs. control, with no difference between them
Nicolau et al., 2022 [39]	Older females (*n* = 43), 66 ± 1 years	NR	Single-blind with four parallel arms: (1) sedentary, (2) sedentary + RSV, (3) PhA, and (4) PhA + RSV	300 mg of RSV daily for 8 weeks	NR	Participants in the PhA and PhA + RSV groups maintained their routine PhA (3 sessions/week, 30 min); no additional exercise was prescribed	↑ resting SBP and DBP in sedentary females vs. exercised peers
Garlic							
Towhidi et al., 2021 [40]	Obese males with HTN (*n* = 50), 53 ± 8 years	NR	Quasi-experimental randomized with five parallel arms: (1) AT + Garlic, (2) AT + PLA, (3) Garlic, (4) PLA, and (5) control	1000 mg of Garlic extract daily for 8 weeks	NR	Treadmill running (3 sessions/week, 50–75% HR_max_, 35–60 min) for 8 weeks	↓ resting SBP and DBP in all groups vs. control, with no difference between them
Kaleh et al., 2022 [41]	Obese females with HTN (*n* = 36), 50–65 years	NR	Double-blind randomized with four parallel arms: (1) AT + Garlic, (2) AT + PLA, (3) Garlic, and (4) control	1000 mg of Garlic daily for 8 weeks	NR	Treadmill running (3 sessions/week, 55–65% HRR, 30–55 min) for 8 weeks	↓ resting SBP in all groups vs. control, with no difference between them ↓ resting DBP only in AT + Garlic group vs. baseline
Ried et al., 2025 [42]	Recreational endurance males and females with elevated arterial stiffness (*n* = 75), 40–65 years	On antihypertensive medication (unspecified)	Double-blind randomized in two parts with two parallel arms, part 1: low dose garlic or PLA, part 2: high dose garlic or PLA	Aged garlic extract for 12 weeks as low dose (2.4 g daily) and high dose (9.6 g daily)	NR	Participants maintained their habitual moderate to high intensity endurance training	↔ resting SBP and DBP in both low and high dose garlic groups vs. PAL after the intervention

ADR: adverse drug reactions, NR: not reported, ACE: angiotensin-converting enzyme, ARB: angiotensin receptor blockers, SBP: systolic blood pressure, DBP: diastolic blood pressure, MAP: mean arterial pressure, AT: aerobic training, RT: resistance training, HIIT: high-intensity interval training, LV-HIIT: low-volume-HIIT, MV-HIIT: moderate-volume-HIIT, PhA: physical activity, 1RM: one-repetition maximum, reps: repetitions, PLA: placebo, HG: handgrip, KE: knee-extensor, HR_max_: maximum heart rate, HRR: heart rate reserve, VO_2peak_: peak oxygen consumption, RPE: rating of perceived exertion, MPO: maximum power output, MVC: maximum voluntary contraction, P_peak_: peak power, CAD: coronary artery disease, HFrEF: heart failure with reduced ejection fraction, HFpEF: heart failure with preserved ejection fraction, EGCG: epigallocatechin gallate, PEH: post-exercise hypotension, T2DM: type 2 diabetes mellitus, CN: curcumin, MetS: metabolic syndrome, Aecc: accentuated eccentric training, Max: maximum, ↑ increase, ↓ decrease, ↔ no change.

## Data Availability

No new data were created or analyzed in this study. Data sharing is not applicable to this article.

## Abbreviations

HTN	hypertension
BP	blood pressure
NO	nitric oxide
BRJ	beetroot juice
GT	green tea
CN	curcumin
RSV	resveratrol
MetS	metabolic syndrome
T2DM	type 2 diabetes mellitus
PAD	peripheral artery disease
CAD	coronary artery disease
COPD	chronic obstructive pulmonary disease
RCT	randomized controlled trial
AT	aerobic training
HIIT	high-intensity interval training
RT	resistance training
1RM	one-repetition maximum
Ppeak	peak power
NO_3_^−^	nitrate
NO_2_^−^	nitrite
SBP	systolic blood pressure
MAP	mean arterial pressure
DBP	diastolic blood pressure
EGCG	epigallocatechin gallate
PEH	post-exercise hypotension
RPP	rate-pressure product
CVD	cardiovascular disease
HRmax	maximum heart rate
sGC	soluble guanylate cyclase
VSMCs	vascular smooth muscle cells
eNOS	endothelial nitric oxide synthase
PI3K/Akt	phosphatidylinositol-3 kinase
AMPK	AMP-activated protein kinase
SIRT1	Sirtuin 1
Nrf2	Nuclear factor erythroid 2 like factor 2
BH4	tetrahydrobiopterin
H2S	hydrogen sulfide
PGC-1α	peroxisome proliferator-activated receptor gamma coactivator 1-alpha
RAAS	renin–angiotensin–aldosterone system
NF-κB	nuclear factor-kappa beta
ACE	angiotensin-converting enzyme
